# Safety of methylprednisolone irrigation during sialendoscopy: effects on the hypothalamic-pituitary-adrenal axis

**DOI:** 10.1007/s00405-025-09678-1

**Published:** 2025-09-23

**Authors:** Konstantinos Garefis, Angelos Chatziavramidis, Dimitrios Goulis, Vasileios Nikolaidis, Konstantinos Markou, Iordanis Konstantinidis

**Affiliations:** 1 2nd Academic ORL, Head and Neck Surgery Department, Aristotle University of Thessaloniki, Papageorgiou Hospital, Thessaloniki, Greece; 2 Unit of Reproductive Endocrinology, 1st Department of Obstetrics and Gynecology, Medical School, Aristotle University of Thessaloniki, Thessaloniki, Greece

**Keywords:** Salivary glands, Sialendoscopy, Ιntraductal ιrrigation, Steroids, Cortisol, Methylprednisolone

## Abstract

**Purpose:**

To assess the safety of methylprednisolone irrigation in the ductal system of salivary glands during sialendoscopy and its impact on the hypothalamic-pituitary-adrenal (HPA) axis.

**Methods:**

Nineteen patients with non-lithiasic sialadenitis were included. Sialendoscopy was performed on the most affected gland (11 parotid, 8 submandibular glands), with 60 mg of methylprednisolone in a 5 ml solution of 0.9% NaCl saline irrigated into the ductal system. Serum cortisol and methylprednisolone concentrations were measured before irrigation, at 2 and 24 h (h) post-irrigation. Patients were examined for post-interventional symptoms and signs 24 h later.

**Results:**

Serum cortisol concentrations increased at 2 h (*p* < 0.05), followed by a decrease (*p* < 0.01) at 24 h. Decrease was recorded in serum cortisol from 0 h to 24 h (17.2 ± 5.9 vs. 9.2 ± 8.0 µg/dl, respectively, *p* < 0.01); 37% of patients fulfilled the criteria for HPA axis suppression (serum cortisol ≤ 1.8 µg/dl). A similar pattern for serum cortisol was observed for the parotid and submandibular glands separately, with more pronounced decrease in the parotid. Serum methylprednisolone increased at 2 h, followed by a decrease to minimal concentrations at 24 h. Pain during methylprednisolone irrigation was reported in 57.9% of patients. Blood pressure and glucose concentrations remained unaffected, and no other symptoms were reported.

**Conclusion:**

Methylprednisolone irrigation during sialendoscopy caused biochemical HPA axis suppression in 37% of patients after 24 h. However, the procedure is considered safe, as there were no reported cases of clinical hypocortisolemia. The decrease in serum cortisol at 24 h was observed mainly after parotid gland irrigation. Minimal drug concentrations were found in the serum after 24 h.

## Introduction

Steroid irrigation into the ductal system of the major salivary glands during sialendoscopy is a common therapeutic practice in chronic sialadenitis with or without stones [1–3]. Its use is sporadic in sialolithiasis cases, depending on the endoscopic findings and interventional technique (e.g., after laser lithotripsy) [3]. In chronic non-lithiasic sialadenitis, steroid use has become part of standard therapeutic protocols. These conditions include recurrent non-lithiasic sialadenitis with or without stenosis, sialadenitis due to Sjögren’s syndrome, juvenile recurrent parotitis and sialadenitis following treatment with radioactive iodine [4–9].

Steroid use as a local application into the ductal system is based on direct action on the epithelial layer of the gland by reducing the inflammatory response and avoiding the adverse effects of systemic administration [4, 10]. However, the local injection of steroids (intra-articularly, subcutaneously for orthopaedic and rheumatologic conditions or epidurally for spine-related and chronic pain conditions) is associated with adverse effects usually seen in systemic administration [11]. Specifically, studies with local injections have shown hypothalamic-pituitary-adrenal (HPA) axis suppression [11–15]. Clinical effects include Cushing syndrome, infection, hyperglycaemia, osteopenia and neuropsychiatric changes, such as temporary mania and psychosis [11]. A variety of steroids have been used during sialendoscopy, such as hydrocortisone, triamcinolone acetonide, prednisone and methylprednisolone [5–9]. The latter is an intermediate-acting compound with a biological half-life of 18–36 h [16].

This pilot study is the first in the literature to assess the safety of methylprednisolone irrigation in the ductal system of the major salivary glands during sialendoscopy and its impact on the HPA axis.

## Materials and methods

### Patients

The study included patients aged > 17 years with recurrent non-lithiasic sialadenitis, Sjögren’s-syndrome-related sialadenitis or sialadenitis following treatment with radioactive iodine or radiation of the head and neck who underwent sialendoscopy with steroid irrigation into the ductal system of the major salivary glands.

Exclusion criteria were lithiasic sialadenitis, steroid intake within the last three months, pregnancy, acute infection, allergy to steroids, hypertension, diabetes mellitus, renal or liver disorders and night shift work. Patients were advised to avoid alcohol for 12 h and food for 3 h before sialendoscopy.

### Methods

 Three blood draws were scheduled within 24 h (baseline at 0 h, 2 h and 24 h). Serum cortisol and methylprednisolone concentrations were measured. Additionally, blood glucose was measured at 0 h and 24 h. The first blood draws and blood pressure measurements were performed at 09:00 (baseline measurement at 0 h). Sialendoscopy was performed under local anaesthesia (4% lidocaine) on the most affected gland. The gland’s papilla was catheterised with dilators of increasing diameter. The sialendoscope (1.1 mm, Erlangen sialendoscope, Karl Storz Co., GmbH, Tuttlingen, Germany) was inserted with simultaneous irrigation with 0.9% NaCl saline solution. The sialendoscope was removed, and the gland was massaged to extrude the administered saline solution before reintroducing the sialendoscope for steroid irrigation. At the end of the procedure, 60 mg of methylprednisolone (Lyo-drol^®^; Vianex, Greece) in a 5-ml solution of 0.9% NaCl saline was rinsed through the sialendoscope.

Patients were instructed to remain fasting and to avoid massaging the gland for the next 2 h; at that time, the second blood draw (2 h) was performed. The following morning at 09:00, the final blood draw (24 h) was conducted, blood pressure was measured and patients were asked if they had experienced any symptoms (yes or no) related to HPA axis suppression, such as fatigue, weakness, dizziness, nausea, vomiting, anxiety or abdominal pain. They were also asked to categorize the local pain separately during saline and methylprednisolone irrigation as mild, moderate, or severe. Patients maintained the same food intake during the post-interventional period to avoid differences in glands’ secretion. The impact of methylprednisolone on the HPA axis was assessed according to morning, fasting serum cortisol concentrations 24 h after methylprednisolone irrigation, with values ≤ 1.8 µg/dl indicating HPA axis suppression [16].

### Ethics

The project was carried out per the Declaration of Helsinki and was approved by the Papageorgiou General Hospital Ethical Committee (Ethics application number: 2023-Β2015 − 257). All patients gave written informed consent to participate in the study.

### Statistical analysis

 The normality of the numerical variables was tested by applying the Shapiro-Wilk test. Age, serum cortisol concentrations, mean glucose concentrations, and blood pressure were presented as means ± standard deviation (SD). Age and serum methylprednisolone concentrations were reported as median with range. To assess the differences in numerical data of the patient group at different time points, the paired t-test was used for parametric variables (serum cortisol concentration, mean glucose concentrations, and blood pressure) and the Wilcoxon-Signed-Rank test for non-parametric variables (serum methylprednisolone concentration). Statistical analysis was performed using the statistical program IBM SPSS Statistics 28.0. The level of significance was set at *p* = 0.05.

## Results

### Demographics

 Nineteen patients met the inclusion criteria and were included in the study. The mean age was 44.9 ± 17.3 years. Of the nineteen patients, eleven (57.9%) were female and eight (42.1%) were male. Five patients were diagnosed with sialadenitis due to Sjögren’s syndrome, two with sialadenitis following radioactive iodine, two following head and neck radiation and ten with recurrent non-lithiasic sialadenitis.

Sialendoscopy was performed in eleven (57.9%) parotid glands and eight (42.1%) submandibular glands **(**Table 1**)**. Of the eleven patients with parotid disease, six (54.5%) were female and five (45.5%) were male. Their median age was fifty-seven years (range: seventeen to seventy). For the submandibular gland, the median age was 30.5 years (range: nineteen to fifty-three). Among the eight submandibular patients, five (62.5%) were female and three (37.5%) were male.

**Table 1:** Study demographics and main results

	All patients	Parotid group	Submandibular group
	*n* = 19	*n* = 11	*n* = 8
Sex *(male/female)*	8/11	5/6	3/5
Age *(years Mean (± SD)/(Range))*	44.9 (± 17.3)	57 (17–70)	30.5 (19–53)
Form of Sialadenitis:			
* Sjögren’s syndrome*	5	4	1
* Radioactive iodine*	2	2	0
* Post radiation*	2	1	1
* Recurrent non-lithiasic*	10	4	6
Serum cortisol 0 h *(µg/dl Mean (± SD))*	17.2 (± 5.9)	17.5 (± 7.4)	16.6 (± 3.2)
Serum cortisol 2 h *(µg/dl Mean (± SD))*	24.5 (± 12.1)	24.0 (± 12.1)	25.2 (± 12.8)
Serum cortisol 24 h *(µg/dl Mean (± SD))*	9.2 (± 8.0)	6.2 (± 6.2)	13.3 (± 8.8)
Serum methylprednisolone 0 h *(µg/dl Median (Range))*	0	0	0
Serum methylprednisolone 2 h *(µg/dl Median (Range))*	0.15 (0.02–0.99)	0.14 (0.02–0.99)	0.20 (0.03–0.78)
Serum methylprednisolone 24 h *(µg/dl Median (Range))*	0.01 (0.00-0.12)	0.01 (0.00-0.03)	0.01 (0.00-0.12)

### Measurements

**Fig. 1:**
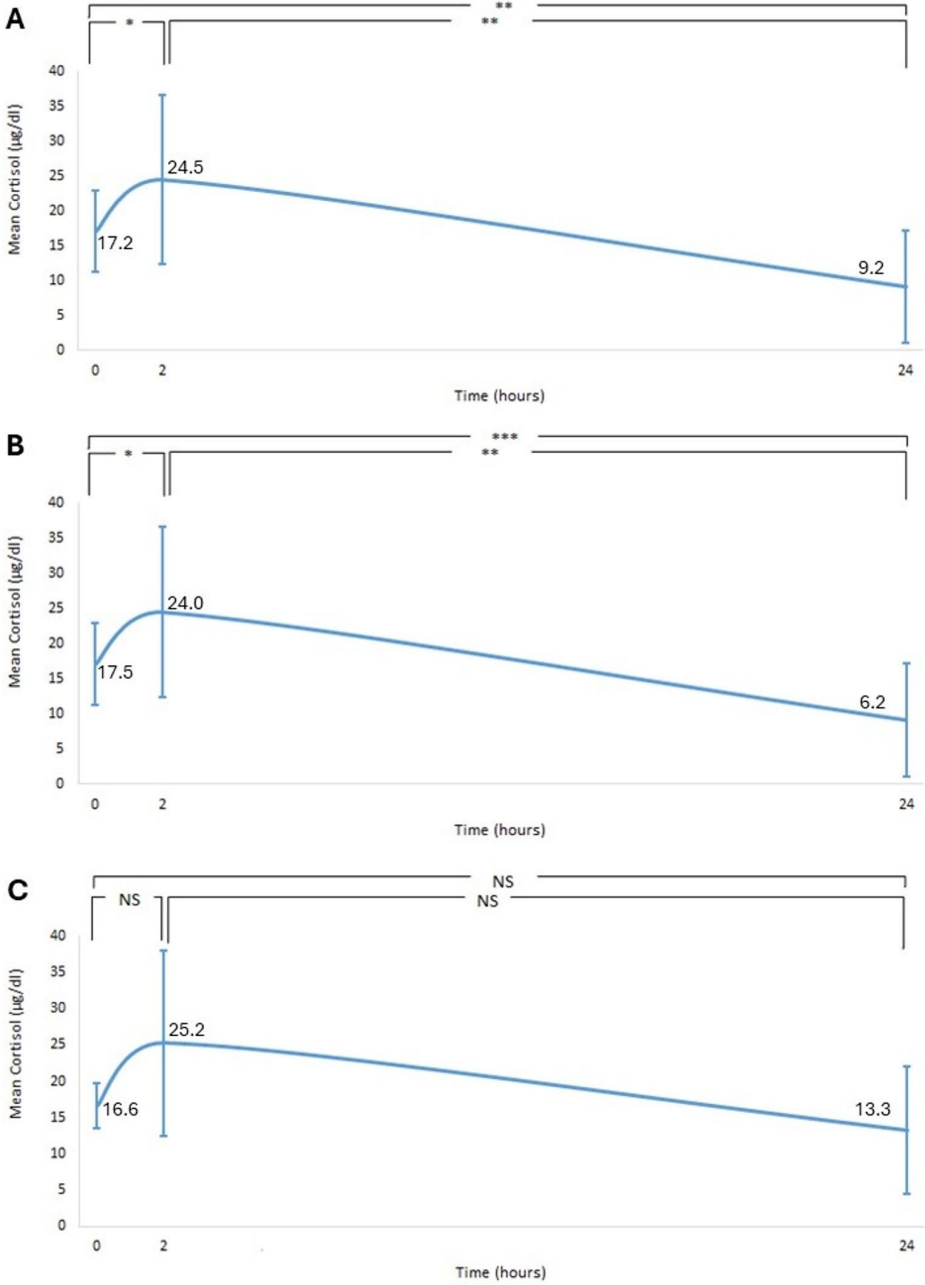
Serum cortisol over 24 h for (**A**) both glands, (**B**) the parotid gland and (**C**) the submandibular gland (* *p* < 0.05, **p < 0.01, *** *p* < 0.001). NS: non-significant

**Fig. 2:**
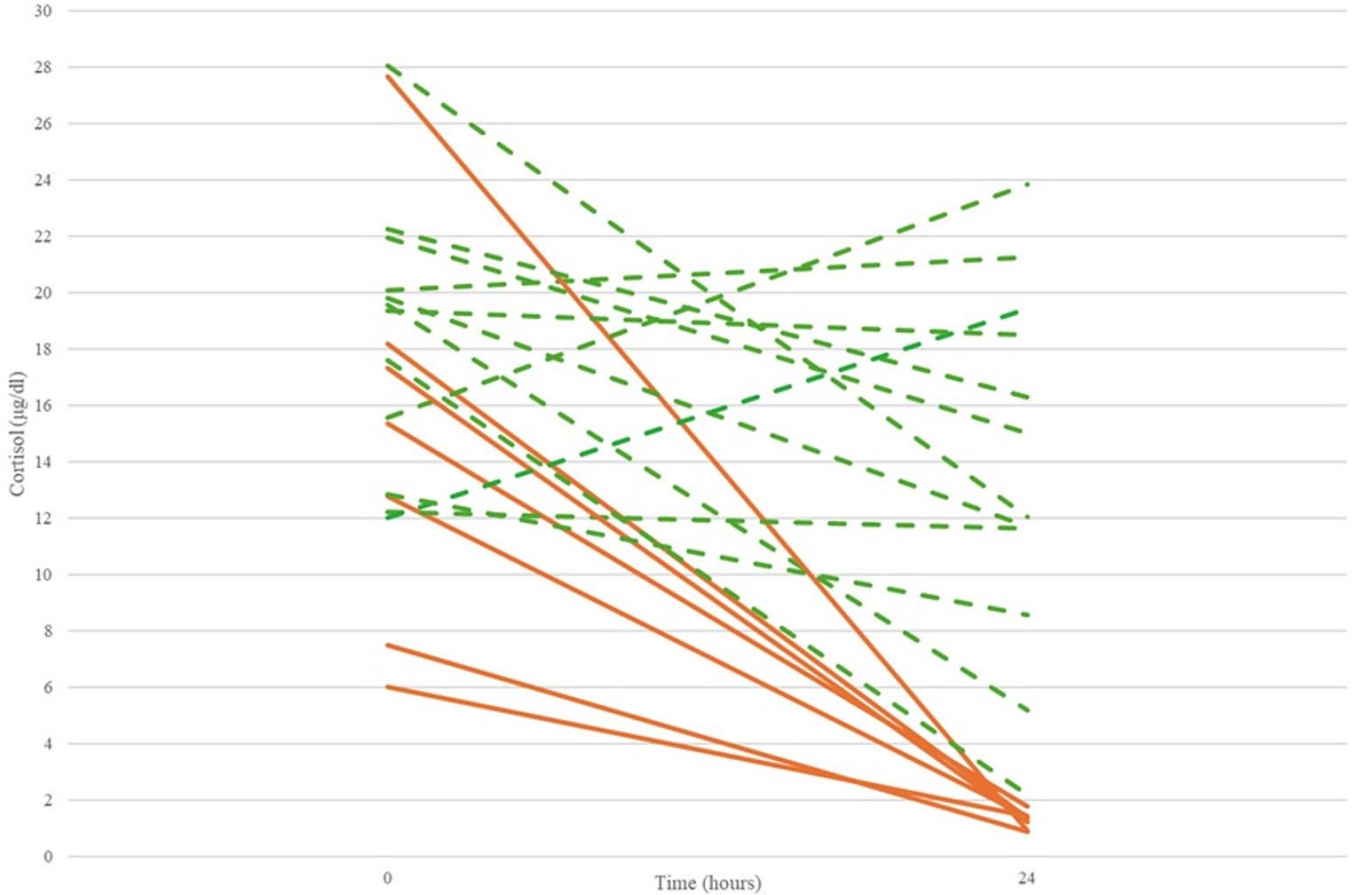
Serum cortisol levels at 0 h and 24 h. Each line corresponds to one patient (Continuous lines: patients with biochemical HPA axis suppression; Non-continuous lines: patients without suppression)

The mean serum cortisol concentration was 17.2 ± 5.9 µg/dl at baseline assessment. At 2 h following steroid irrigation, cortisol concentrations increased to 24.5 ± 12.1 µg/dl (*p* < 0.05). From 2 h to 24 h post-sialendoscopy, cortisol concentrations decreased to 9.2 ± 8.0 µg/dl (*p* < 0.01) (Fig. 1A). From 0 h to 24 h, serum cortisol concentration decreased in 73.4% of patients (14 out of 19) by 45.5% (*p* < 0.01), while 26.3% (5 out of 19) showed no change. In addition, 37% of patients (7 out of 19) fulfilled the criteria for HPA axis suppression (serum cortisol ≤ 1.8 µg/dl) (Fig. 2).

**Fig. 3:**
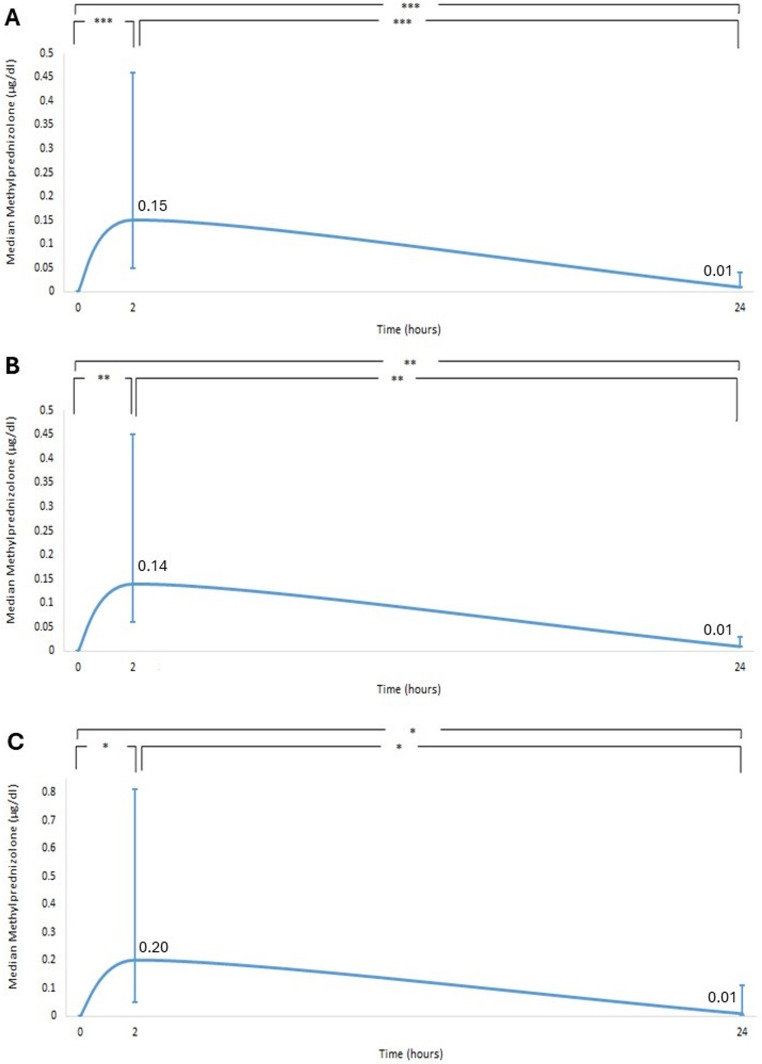
Serum methylprednisolone over 24 h for (**A**) both glands, (**B**) the parotid gland and (**C**) the submandibular gland (* *p* < 0.05, **p < 0.01, *** *p* < 0.001)

Serum methylprednisolone concentrations were 0 µg/ml at baseline with an increase at 2 h (median: 0.15 µg/ml, range: 0.02–0.99) (*p* < 0.001). They decreased from 2 h to 24 h (median: 0.01 µg/ml, range: 0.0013–0.124) (*p* < 0.001) (Fig. 3A).

Eight out of 19 patients (42%) experienced mild pain during saline irrigation in the parotid or submandibular area. Nine patients (47.4%) experienced moderate pain, and two patients (10.5%) experienced mild pain during methylprednisolone irrigation, while the rest remained asymptomatic.

Mean glucose concentrations at 0 h (95.3 mg/dl ± 12.5) did not differ from those at 24 h (95.1 mg/dl ± 8.7) (*p* > 0.05). Similarly, blood pressure did not change from 0 h (122.6 ± 15.8 vs. 76.1 ± 7.0 mmHg) to 24 h (121.5 ± 13.2 vs. 73.8 ± 6.5 mmHg) (*p* > 0.05). Since no changes in glucose concentrations or blood pressure were recorded, and no other symptoms related to HPA axis suppression—such as fatigue, weakness, dizziness, nausea, vomiting, anxiety, or abdominal pain—were reported, no cases were considered as clinical hypocortisolemia.

### Parotid gland/Submandibular gland

The mean serum cortisol concentrations in the parotid and submandibular glands followed a similar pattern, increasing at 2 h and decreasing at 24 h. However, in the submandibular gland, there were no significant differences between time points (*p* > 0.05), as observed in the parotid gland (Fig. 1B and C). Serum cortisol concentrations decreased from 0 h to 24 h and were more pronounced in the parotid (from 17.5 ± 7.4 to 6.2 ± 6.2 µg/dl, 65.9% decrease from baseline) than the submandibular gland (from 16.6 ± 3.2 to 13.3 ± 8.8 µg/dl, 17.4% decrease from baseline). Among patients with parotid gland disease, 9.1% (1 out of 11) showed no changes in cortisol concentrations from 0 h to 24 h. In contrast, among patients with submandibular gland disease, 50% (4 out of 8) showed no changes. Specifically, regarding HPA axis suppression, 46% of patients with parotid gland disease (5 out of 11) met the criteria for HPA axis suppression (serum cortisol ≤ 1.8 µg/dL), while 25% (2 out of 8) of patients with submandibular gland disease did. The serum methylprednisolone concentrations in each gland followed a similar pattern for all patients (Fig. 3B and C).

### Underlying pathology

Serum cortisol and methylprednisolone concentrations were categorized according to the underlying pathology into three groups: autoimmune background, patients who had undergone irradiation, and those with chronic non-lithiasic sialadenitis (Table 2). Statistical analysis was not feasible due to the small number of patients in each subgroup. Serum cortisol and methylprednisolone concentrations appeared to be approximately similar across the three groups at various time points.

**Table 2:** Results according to the underlying pathology

Form of Sialadenitis	All	Autoimmune	After irradiation	Chronic non-lithiasic
Number of patients	19	5	4	10
Serum cortisol 0 h *(µg/dl Mean (± SD))*	17.2 (± 5.9)	16.6 (± 8.2)	22.5 (± 3.9)	15.3 (± 4.1)
Serum cortisol 2 h *(µg/dl Mean (± SD))*	24.5 (± 12.1)	20.4 (± 10.1)	29.8 (± 13.5)	23.6 (± 10)
Serum cortisol 24 h *(µg/dl Mean (± SD))*	9.2 (± 8.0)	6.3 (± 5.3)	8.4 (± 8.3)	11 (± 9.2)
Serum methylprednisolone 0 h *(µg/dl Median (Range))*	0	0	0	0
Serum methylprednisolone 2 h *(µg/dl Median (Range))*	0.15 (0.02–0.99)	0.1 (0.02–0.28)	0.34 (0.07–0.99)	0.2 (0.03–0.78)
Serum methylprednisolone 24 h *(µg/dl Median (Range))*	0.01 (0.00-0.12)	0.01 (0.00–0.01)	0.01 (0.00–0.03)	0.01 (0.00–0.12)

## Discussion

This pilot study is the first to assess the safety of methylprednisolone irrigation in the ductal system of the major salivary glands during sialendoscopy and its impact on the HPA axis. This study has four main conclusions:

1. Biochemically confirmed HPA axis suppression was observed 24 h after rinsing the ductal system of the major salivary glands with a 60-mg methylprednisolone solution, as 37% of patients fulfilled the criteria for HPA axis suppression.
2. The decrease in serum cortisol concentrations was more pronounced in the parotid gland (65.9%) than in the submandibular gland (17.4%). Biochemically confirmed HPA axis suppression was observed in 46% of patients with parotid gland disease and 25% of patients with submandibular gland disease.
3. Blood methylprednisolone concentrations reached minimal concentrations 24 h after the drug’s administration.
4. Methylprednisolone irrigation is considered safe within the first 24 h as no clinically significant signs or symptoms were observed.

No study in the literature evaluates the effect of methylprednisolone irrigation during sialendoscopy on the HPA axis. The primary actions of steroids in the HPA axis are to lower blood cortisol and adrenocorticotropic hormone [11–17]. Following steroid administration, cortisol suppression is a sign of systemic absorption [11, 18, 19]. Studies involving intra-articular or epidural steroid injections report partial HPA axis suppression [11–15]. The duration and extent of HPA axis suppression varies from a few days to four weeks, depending on the type of drug administered, drug solubility, dose, number of injections and injection site, as well as the patient’s metabolic, thyroid, liver and renal function and comorbidities [11, 12, 15, 20, 21]. In our study, the increase in serum cortisol concentrations at 2 h after irrigation was most likely due to patient stress during the sialendoscopy and the pain caused by the irrigation [22]. The 45.5% decrease at 24 h is likely attributable to the effect of methylprednisolone. However, this decrease was not uniform: 23.6% of patients showed no change or even an increase in serum cortisol concentrations following methylprednisolone irrigation. In contrast, in 37% of patients serum cortisol at 24 h was below the 1.8 µg/dL threshold required for biochemical HPA axis suppression [16]. Regarding the difference in serum cortisol concentrations after methylprednisolone irrigation between the parotid and submandibular glands, there are currently no studies in the literature that propose or justify potential pathophysiological mechanisms.

There are various ways that steroids can get into saliva. Charged steroids, like dehydroepiandrosterone sulfate, diffuse between the tight junctions of acinar cells, and their concentrations are inversely related to saliva flow rate. In contrast, neutral steroids, such as methylprednisolone, diffuse directly through the acinar cells, so their concentrations remain unaffected by saliva flow rate [23]. Additionally, salivary pH, which fluctuates with flow rate, influences the absorption of several steroids [24]. Steroids reduce pro-inflammatory cytokines, such as tumour necrosis factor and interleukin-1b, and support the restoration of salivary gland function in inflammatory conditions, particularly those resulting from obstruction due to fibrosis. This effect is achieved by modulating the activity of macrophages and myofibroblasts [25]. Treatment responses to steroids also vary depending on the underlying disease. For example, in radiation-induced sialadenitis, both luminal ductal and acinar cells are affected. Luminal ductal cells, which express Na⁺/I⁻ symporters, are the primary targets in radioiodine-induced sialadenitis due to their active uptake of radioactive iodine. Acinar cells are likely affected due to disruptions in calcium signaling, increased production of reactive oxygen species, or DNA damage. Additionally, acinar cells may be damaged because the K⁺/Na⁺/Cl⁻ transporter system, essential for fluid secretion, is located in these cells. This may explain why the parotid glands are more frequently involved [26–29]. However, when our patients were categorized according to the underlying pathology, similar serum cortisol and methylprednisolone concentrations were observed among the three subgroups **(**Table 2**)**. This may be due to the limited number of patients in each subgroup.

Systemic absorption of methylprednisolone from the ductal system of the salivary glands could not be avoided, as demonstrated by its detection in the blood. The difference (*p* < 0.001) in methylprednisolone concentrations between 0 h and 24 h was largely due to the zero variability that patients had at 0 h, making the assay highly sensitive to small changes. The absolute change of 0.02 µg/ml is clinically negligible, falling within the range of normal variation or measurement error. Thus, methylprednisolone concentrations at 24 h can be practically interpreted as the same as baseline concentrations. Systemic absorption may vary between the steroids, likely based on their solubility [11, 15]. For example, in intra-articular injections, the duration of local and systemic actions is longer for less soluble steroids [30]. In our study, methylprednisolone was immediately absorbed into the systemic circulation, as detected in the serum concentrations 2 h after irrigation. Systemic absorption likely occurred directly through the ductal system of the major salivary glands and from the oral mucosa as the drug escaped from the ductal system into the oral cavity over 24 h. The decrease in methylprednisolone serum concentrations at 24 h was likely due to its intermediate half-life [16]. Unlike injections into closed cavities (e.g., intra-articular), the function of the salivary glands produces a continuous saliva flow, leading to a faster washout of the drug.

No symptoms suggestive of adrenal insufficiency were reported by patients 24 h after sialendoscopy. Cases of secondary Cushing syndrome have been reported following intra-articular administration of steroids. Hyperglycaemia, negative effects on bone metabolism and bone density, immunosuppression and neuropsychiatric disorders have also been documented after intra-articular or epidural steroid injections [11]. The absence of symptoms related to secondary adrenal insufficiency is likely due to the action of glucocorticoids mediated by methylprednisolone, the minimal serum methylprednisolone concentrations at 24 h, and the drug’s intermediate duration of action [16]. Since sialendoscopy is a non-invasive and well-tolerated procedure, an intermediate half-life steroid could be administered more frequently to relieve patients’ symptoms.

Steroid irrigation during sialendoscopy is an effective method, and although it has been used for decades, there are no reports of clinical hypocortisolemia [1, 5, 6, 9, 22, 27, 31]. This aligns with our results, as none of our patients exhibited symptoms related to HPA axis suppression. However, clinicians should be cautious with steroid irrigation during sialendoscopy, particularly in high-risk patients, such as postmenopausal women, those with diabetes mellitus or hypertension or those scheduled for surgery [11]. Systemic absorption in patients not considered high risk indicates that further studies are needed for these populations.

This study had several limitations, such as the relatively small number of patients and the need for a control group (patients treated with saline irrigation only). However, as this is a pilot study, further research with larger patient populations is needed to confirm the safety of steroid irrigation during sialendoscopy and its effects across various glands and pathologies. The lack of serum cortisol measurements in the days following the procedure until full recovery of the HPA axis, along with adrenocorticotropic hormone measurements, constitute additional limitations. Lastly, the study did not employ a more detailed pain assessment tool, such as the Visual Analog Scale, which may affect the reliability and comparability of patient-reported pain outcomes.

## Conclusion

Methylprednisolone irrigation during sialendoscopy resulted in biochemically confirmed HPA axis suppression in 37% of patients after 24 h. Serum cortisol concentrations decreased at 24 h, mainly after parotid gland irrigation. Additionally, 24 h after methylprednisolone irrigation, only minimal drug concentrations were found in the patients’ serum. This procedure can be considered safe, as no cases of clinical hypocortisolemia were recorded, and no adverse effects were reported. However, randomized clinical trials are necessary to establish the safety of steroid use during sialendoscopy.

## Data Availability

All data for this study is presented in this paper.
